# Bioactive Nutrient Fortified Fertilizer: A Novel Hybrid Approach for the Enrichment of Wheat Grains With Zinc

**DOI:** 10.3389/fpls.2021.743378

**Published:** 2021-12-23

**Authors:** Muhammad Asif Ali, Farrukh Naeem, Nadeem Tariq, Ijaz Ahmed, Asma Imran

**Affiliations:** ^1^Engro Fertilizers Ltd., Lahore, Pakistan; ^2^First Biotech LLC, Lahore, Pakistan; ^3^National Institute for Biotechnology and Genetic Engineering-Campus-Pakistan Institute for Engineering and Applied Sciences (NIBGE-C-PIEAS), Faisalabad, Pakistan

**Keywords:** zinc-biofortification, Zn solubilizing bacteria, bio-fortified nutrient fertilizer, plant-growth, rhizosphere, agriculture productivity

## Abstract

Zinc (Zn) is a critical micronutrient that synergizes nutrient use efficiency, and improves plant growth and human health. Low Zn bioavailability in soils affects produce quality and agricultural productivity worldwide ultimately inducing deficiency in humans and animals. Zn deficiency is a leading cause of malnutrition in underdeveloped countries where a widespread population depends upon staple cereals for daily intake of calories. Modern cereal cultivars are inherently low in Zn, eventually, plants need to be enriched with soil application of ZnSO_4_, but due to higher fixation losses, it becomes an inefficient source. Rhizosphere microbiome contains Zn-solubilizing bacteria (ZSB) that improve Zn bioavailability, thus increase the root function, Zn uptake, and plant growth. Niha Corp developed a hybrid process of bioactive nutrient fortified fertilizer (BNFF), which has been used to formulate Zabardast Urea (ZU) by coating bioactive Zn (BAZ) and ZSB on urea. Data obtained for 15 wheat varieties from 119 farmer field demonstration plots and eight replicated trials on 42 locations across multi-environment conditions conclude that ZU significantly improved the plant biomass and yield by 12% over non-Zn control and produced grains with 57 μg/g Zn contents, which can meet a major part of the recommended dietary allowance (RDA) of humans. The study recommends that this microbe-mediated hybrid invention (ZU) is a feasible approach to boost Zn bioavailability and Zn use efficiency, with enhanced yield and quality that may contribute to improve human health. To the best of our knowledge, this is the first wide-scale field testing of Zn enrichment in the grains of bread wheat using an innovative BNFF Urea Z technology.

## Highlights

- Millions of people around the globe suffer from Zn deficiency-related growth and physiological disorders.
- A plant-based diet (e.g., cereals) is inherently low in Zn content and also contains Zn inhibitors (e.g., phytates) that reduce the Zn availability for human consumption.
- Zn biofortification of cereals can reduce the problems related to Zn-deficiency disorders.
- We reported a novel approach based on bioactive nutrient fortified fertilizer (BNFF) to increase the Zn uptake in the wheat grain making it fit for human consumption.
- The technology can be used at a large scale as it eases the application of BAZ along with beneficial microbes coated on urea for easy application.

## Introduction

Zinc (Zn) is an important micronutrient for cellular, physiological, and biological growth and development (Cakmak and Kutman, 2018). As a metallic cofactor, it activates and stabilizes more than 300 enzymes in plants, animals, and humans (McCall et al., 2000; Gurmani et al., 2012; Lacerda et al., 2018), mainly regulating vital processes, including DNA and protein synthesis, carbohydrate metabolism, gene expression, enzyme activation, photosynthesis, hormones, disease resistance, wound healing, and fertility. Zn finger proteins (ZFP) are considered unique due to their synergetic role during phytohormone response, plant growth, and development (Nauman et al., 2018). Many Zn-dependent enzymes are involved in carbohydrate metabolism, especially in leaves, and affect proteins, auxins, and membrane integrity. Hence, its deficiency in plants seriously distresses various vital processes leading to yield losses and lower Zn contents in grains and fruits (Imran et al., 2015; Xing et al., 2016).

Zinc is the fourth essential micronutrient controlling a number of proteins in humans (Cakmak, 2008; Shivay et al., 2015; Krezel and Maret, 2016). It plays a major role in the function of the brain, immune system, and endocrine system, and its deficiency is connected to many physiological and growth disorders, such as premature death, stunted growth, underweight children, poor appetite, delayed healing, taste abnormalities, blindness, cognitive losses, or mental lethargy (FAO, 2004, 2021; Bhutta et al., 2007; Khalid et al., 2014; Sauer et al., 2016). A direct positive correlation has been observed for serum Zn level with the development of diabetes (Anjum et al., 2012), depressive disorders, and bipolar depression (Cope and Levenson, 2010). An estimated 2.7 billion global population is Zn deficient, while further ∼50% of the population are at risk (WHO/FAO, 2006) mainly due to low dietary intake and consumption of cereal-based foods, which are naturally low in Zn contents and contain Zn-absorption inhibitors, e.g., phytic acid (Hambidge et al., 2011). Global data analysis reveals that wheat grain contains 31.8 μg g^–1^ of Zn (Wang et al., 2020), but its absorption and efficacy depend upon the intake quantity, the milling and fermentation practices, and Zn or phytate intake from other food sources (Brown et al., 2010). The optimum level of grain Zn should be 40–50 μg g^–1^ to meet recommended dietary allowance (RDA), which is 11 mg for men and 8 mg for women (Bouis et al., 2011; Chen et al., 2017; Cakmak and Kutman, 2018).

Insufficient Zn in the rhizosphere leads to Zn-deficient grains as the rhizosphere is the site from where Zn moves to roots and shoots and later accumulated in grains (White and Broadley, 2011; Maillard et al., 2015). Almost 50% of the world soils under cereal production are Zn deficient (Welch et al., 2013). Many factors, e.g., texture, pH, water content, organic matter, the concentration of calcium carbonate, basic cations (Na, Ca, and Mg), dissolved organic carbon (DOC), and cation exchange capacity, anions bicarbonates, and phosphates, influence the Zn transformation and bioavailability in soil (Alloway, 2009; Koshgoftarmanesh et al., 2018). In calcareous soils, total Zn may be relatively large, but low organic matter and higher calcium carbonate contents reduce its availability (Bityutskii et al., 2017; Chen et al., 2019). Various soil supplements are being used to increase rhizosphere Zn bioavailability (Noor-ul-Ain, 2019). Zinc sulfate (ZnSO_4_) is the most common fertilizer with high solubility; however, it readily undergoes fixation, which reduces its availability (Hussain et al., 2015). Zinc oxide (ZnO) is also not recommended due to very low solubility (Ahmad et al., 2018). Furthermore, crop Zn use efficiency is very low, i.e., 4–8% (Shivay et al., 2008), which needs to be improved either by Zn biofortification or by improving and economizing the available Zn-fertilizer sources (Montalvo et al., 2016). Zn-solubilizing bacteria (ZSB) are known components of the rhizosphere playing a significant role in nutrient availability and transformation (Hakim et al., 2021). ZSB convert the unavailable forms of Zn to plant-available forms and increase its uptake and accumulation in grains (Mishra et al., 2017). These bacteria are usually versatile, hence, exert synergistic-beneficial impact on plant growth and yield (Imran et al., 2021). Application of ZSB as biofertilizers has been reported in wheat and maize (Goteti et al., 2013; Kamran et al., 2017; Hussain et al., 2019), but its field application needs extra time and effort as biofertilizers are applied separately from the chemical fertilizers. Combining fertilizers (i.e., biological and chemical) into a single product, however, is a better approach to save time, effort, and cost.

Bioactive nutrient fortified fertilizer (BNFF) is a novel concept developed and patented recently in the fertilizer industry (Tariq et al., 2017). The BNFF is a patent of Niha Crop United States where it is prepared by organic encapsulation of bioactive nutrients (P, Zn, Fe, etc.) and beneficial microbial consortium with subsequent coating onto chemical urea fertilizer. BNFF serves as a rich source of beneficial microbial strains, which not only improves nutrients use efficiency, induces resistance in plants, but also provides growth-regulating organic nutrients for healthy growth that leads to higher yield and better-quality yield. The consortium of beneficial microbes solubilizes a range of nutrients present in the root zone. It also facilitates extensive root system development and activates the inherent defense response of plants through induced systemic resistance (ISR) (Choudhary et al., 2007). Production and implication of bioactive organic fertilizer enriched with ZSB have been reported to boost Zn contents in maize at a small scale (Hussain et al., 2020). The present study reports a novel hybrid technology that combines the benefits of biological (ZSB) and chemical fertilizers (BAZ + urea) with a synergistic effect on the plant at a wide scale. Although, the effects of ZSB (Khanghahi et al., 2018; Eshaghi et al., 2019) and urea have been tested in solo treatments.

This study hypothesized that bioactive zinc (BAZ) will keep Zn free from getting fixed in the soil due to the protective cover of organic encapsulation, therefore, Zn will remain continuously available in the root zone for plant uptake throughout the crop life. The ultimate aim of the study was to use BNFF to fortify wheat grains with Zn to reduce Zn deficiency in humans. This study reports and confirms the potential of BAZ fortified urea to improve physiology, growth, yield, the concentration of Zn in grains, and ultimately the grain quality of different wheat varieties grown at farmer fields for human consumption. To the best of our knowledge, this is the first report of the development and large-scale testing of BAZ for bread wheat biofortification.

## Methodology

### Materials Used

Bioactive Zn (BAZ) was formulated using the patent method of Niha Corp, Ontario, CA, United States as a fine dry powder of biologically solubilized and organically encapsulated Zn (Tariq et al., 2017). BNFF Urea Z was developed by First Biotech LLC, Lahore, Pakistan an associate of Niha Corp., United States, using the patented process (Tariq et al., 2017). Engro Fertilizers Ltd. (EFERT), having exclusive marketing rights of BNFF Urea Z for Pakistan, launched under the brand name Zabardast Urea (ZU) in 2017. ZU contains 42% nitrogen, 1% BAZ, and a consortium of beneficial microbes 10^3^ CFU g^–^1 of ZU material. The beneficial microbes are a consortium of Zn-mobilizing bacteria with multiple plant benefits (Tariq et al., 2017).

### Field Location, Soil Analysis, and Experimental Design

Cross-ecological trials were carried out during the wheat season 2019–2020 to evaluate the ZU application on the yield and quality of 15 bread wheat (*Triticum aestivum*) varieties. Eight replicated trials were conducted spread over eight different sites (i.e., Shujabad, Multan, Chichawatni, Lahore, Sahiwal, Faisalabad, Kasur, and Chiniot) on farmer fields; in addition, 119 demonstration trials were conducted at 42 different sites. Random soil sampling was done at a depth of 0–20 cm. Samples were pooled, homogenized, air-dried, sieved (2 mm), and characterized for different parameters such as pH, EC, available phosphorous and potassium (US Salinity Laboratory Staff, 1954; Page et al., 1982), and extractable metal (Zn) (Soltanpour, 1985). The soil data are presented in Supplementary Table 1.

The treatment plan at replicated field trials at eight locations is as follows:

T1: Urea + no Zn control [urea; 46% N @ 250 kg ha^–1^]
T2: Urea + Zn sulfate [urea; 46% N @ 250 kg ha^–1^) + Zn sulfate non-branded (33% Zn @ 15 kg ha^–1^)
T3: Urea + Zingro (urea; 46% N @ 250 kg ha^–1^) + Engro brand Zn fertilizer [Zingro; 33% Zn @ 15 kg ha^–1^]
T4: BNFF Urea Z (ZU) (urea (46% N) 125 kg + ZU (42% N) 125 kg ha^–1^, 1% bioactive Zn (BAZ) (1.235 kg ha^–1^) and 10^3^ CFU g^–1^ beneficial microbial consortium].

The layout of the field experiment was RCBD split-plot design with treatments as main plots and variety as subplots with three replicates at each location. The plot size per replicate was 252.9 m^2^.

For 119 farmer field trials at 42 locations, the treatments were as follows:

T1: Farmer practice: urea + Zn sulfate [urea; 46% N @ 250 kg ha^–1^) + Zn sulfate non-branded (33% Zn @ 15 kg ha^–1^).
T2: BNFF Urea Z (ZU) (125 kg urea (46% N) + 125 kg ZU (42% N) ha^–1^, 1% BAZ (1.235 kg ha^–1^), and 10^3^ CFU g^–1^ beneficial microbial consortium].

The plot size at farmer sites was 0.5 acres (2,023.4 m^2^) per treatment for each trial.

Sowing was performed from November 15 to December 15, 2019. Uniform application of nitrogen, phosphorus, and potassium was done across all treatments at the rate of 250, 185, and 150 kg ha^–1^, respectively. Diammonium phosphate (DAP) was used as a phosphorus source and mutate of potash (MOP) as a potassium source, while the use of nitrogen and Zn varied as explained above in the treatment plan. All P and K fertilizers were applied at the time of sowing, whereas nitrogen was applied in two splits, i.e., half 25 days after sowing and the remaining half 55 days after sowing using the broadcast method followed by flood irrigation. In ZU-treated plants (T4), the ZU was applied during the first split dose, while normal urea was applied in the second dose. The plants were irrigated with canal water as and when required.

### Growth and Yield Analysis

Productive tillers (m^–2^) were determined 70 days after sowing as an average of three random locations from each replicate plot using a 1 m^–2^ steel template. A sample of 15 random plants was selected from each treatment at each location for data readings related to growth and yield at harvest (125 days after sowing). Plant height and spike length were measured using a meter scale. Shoot fresh biomass was recorded for a random sample of 15 plants of each treatment (5 from each replicate) at all locations. The number of grains per spike was determined from 15 spikes collected randomly from the sample of 15 plants of each treatment (5 plants per replicate plot). After recording aforesaid data, plant samples were threshed and 1,000 kernel weight was determined by weighing 100 grains of three sets, then multiplying their average by 10, and recorded for each treatment, accordingly. For final yield data, the wheat crop of each plot was harvested, threshed, weighed, and recorded for grains and straw, separately for statistical analysis. The farmer field trial data were harvested from the whole plot and yield was determined.

#### Analysis for Zinc in Grains

Three random samples of wheat grains (5 g each) were collected from each threshed produce of each treatment. The seeds were washed using distilled water and then air-dried without exposure to direct sunlight followed by oven drying (65°C for 72 h), separately. These samples were finely ground in a grinder (IKA WERKE, MF 10 Basic, Staufen, Germany) and wet digested in a diacid mixture (HNO_3_:HClO_4_ ratio of 2:1) (Jomes and Case, 1990). The Zn concentration was measured in the digest by an atomic absorption spectrophotometer (PerkinElmer, Analyst 100, Waltham, United States). Agronomic Zn use efficiency (ZUE) was calculated as described by Zulfiqar et al. (2020):


$$\mathrm{Agronomic}\mathrm{efficiency}\left(\text{AgE}\right)=\frac{{\text{GY}}_{\text{Zn}}-{\text{GY}}_{\text{C}}}{{\text{Zn}}_{\text{a}}}$$

where GY_*Zn*_ is the grain yield of Zn-treated plots, GY_*C*_ is the yield of untreated plots, and Zn_*a*_ is the amount of Zn applied.

### Statistical Analysis

This was a random sample, farmer field trial study that precisely detected the differences in the varietal response within and across the environments. Engro field trial data at eight different sites were subjected to analysis of variance using computer software Statistix version 8.1 (Analytical Software, United States). The treatment data were averaged over locations to calculate the mean response of ZU across the cross-ecological trials. The treatment means were compared using the least significant difference test (Steel et al., 1997) at a 5% probability level. Similarly, the farmer field data were averaged over varieties and 125 locations to analyze the varietal response. Correlation analysis, regression, and principal component analyses were performed using IBM SPSS software for Windows (SPSS Version 20, NY, United States).

## Results

### Productive Tillers and Spike Length (cm)

**FIGURE 1:**
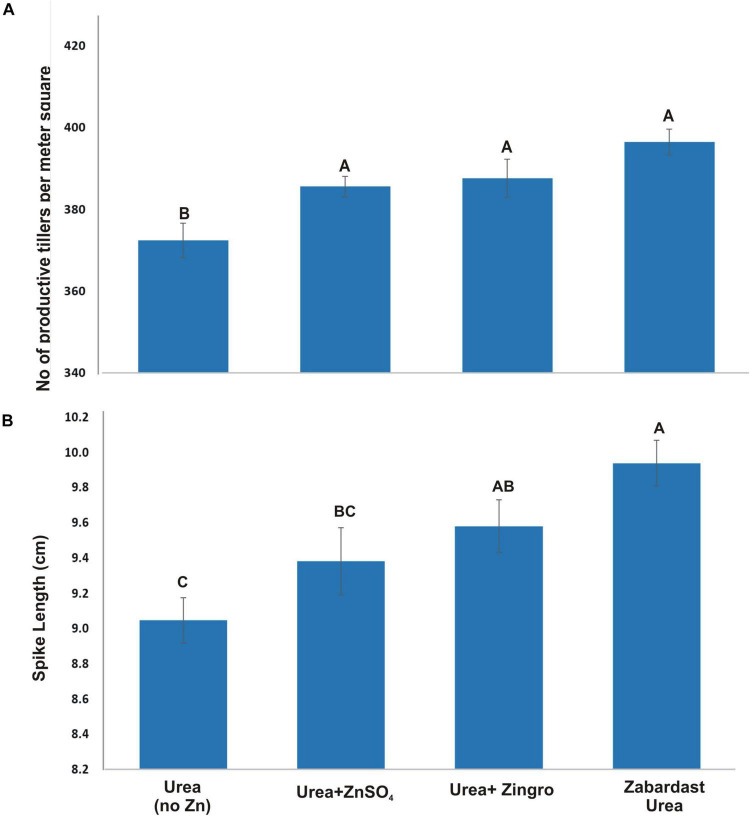
Effect of ZU application on the productive tillers **(A)** and spike length **(B)** of wheat in the field compared with simple urea and Zn treatments. The data are an average of 8 replicated trials (averaged over locations and varieties).

Statistical analysis of data obtained from field trials at eight locations revealed that the application of ZU produced the highest number of productive tillers as compared to other Zn treatments and non-Zn controls (Figure 1A). The average productive tillers were significantly high in ZU-applied plots (396) with a percentage increase of 6.45 over non-Zn control (372). However, the productive tillers with Zingro (388) showed an increase of 4.30 and with ZnSO_4_ (386) a 3.5% increase over non-Zn controls. The ZU-treated plots also produced the longest spikes (9.9 cm) as compared to non-Zn control (9.0 cm) and other sources of ZnSO_4_ (Figure 1B). The percentage increase by ZU was 9.9%, followed by Zingro and ZnSO_4_ treatments with 5.9 and 3.7% increase, respectively, over non-Zn controls.

### Number of Grains per Spike and 1,000 Grain Weight (g)

**FIGURE 2:**
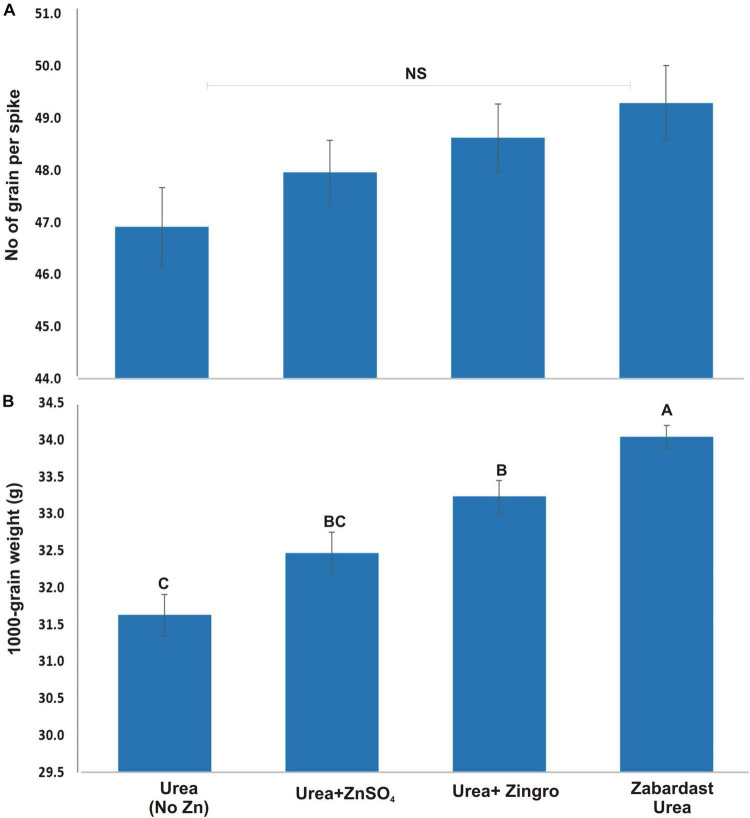
Effect of ZU application on the number of grains per spike **(A)** and 1,000 grain weight **(B)** of wheat in the field compared with simple urea and Zn treatments. The data are an average of 8 replicated trials (averaged over locations and varieties).

The number of grains per spike showed a statistically non-significant effect (Figure 2A) by different Zn application treatments from field trials at eight locations. On average, ZU application showed maximum grains per spike (49.3) followed by Zingro (48.6) and ZnSO_4_ (48.0) compared with non-Zn controls (46.9). The 1,000 grain weight, however, showed a significant treatment response where maximum grain weight was observed in ZU-treated plots (34.0 g) followed by Zingro (33.2 g) and ZnSO_4_ (32.5 g) compared with non-Zn controls (31.6 g) (Figure 2B). The percentage increase in grain weight was 7.5 with ZU treatment, 3.6 with Zingro, and 2 with ZnSO_4_ over non-Zn controls.

### Total Biomass, Grain Yield, and Straw Yield

**FIGURE 3:**
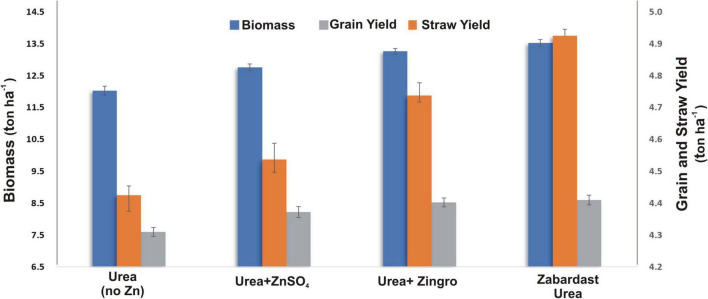
Effect of ZU application on total biomass, grain yield, and straw yield of wheat in the field compared with simple urea and Zn treatments. The data are an average of 8 replicated trials (averaged over locations and varieties).

The total biomass obtained from eight field trials at eight locations (Figure 3) showed a significant increase by the application of ZU (13.5 t ha^–1^) with a percentage increase of 12 over non-Zn controls (12.0 t ha^–1^). This was followed by other Zn treatments, i.e., Zingro (13.3 t ha^–1^), and ZnSO_4_ (12.8 t ha^–1^) with an increase of 10 and 6%, respectively, over non-Zn control.

All Zn treatments generally exhibited a positive impact on the grain yield (Figure 3), but comparative analysis showed that ZU treatment produced the maximum grain yield (4.9 t ha^–1^), where the percentage increase over control was 11.7% followed by Zingro (4.7 t ha^–1^), and ZnSO_4_ (4.5 t ha^–1^) with an increase of 7.2 and 2.8% over control, respectively. A similar trend was observed for the data obtained for the straw yield (Figure 3) where the maximum yield (8.6 t ha^–1^) was obtained in the ZU plots with an increase of 12.6%, followed by Zingro (yield: 8.5 t ha^–1^; percentage increase 11.8%) and ZnSO_4_ (yield: 8.2 t ha^–1^; percentage increase 8.0%).

### Harvest Index

**FIGURE 4:**
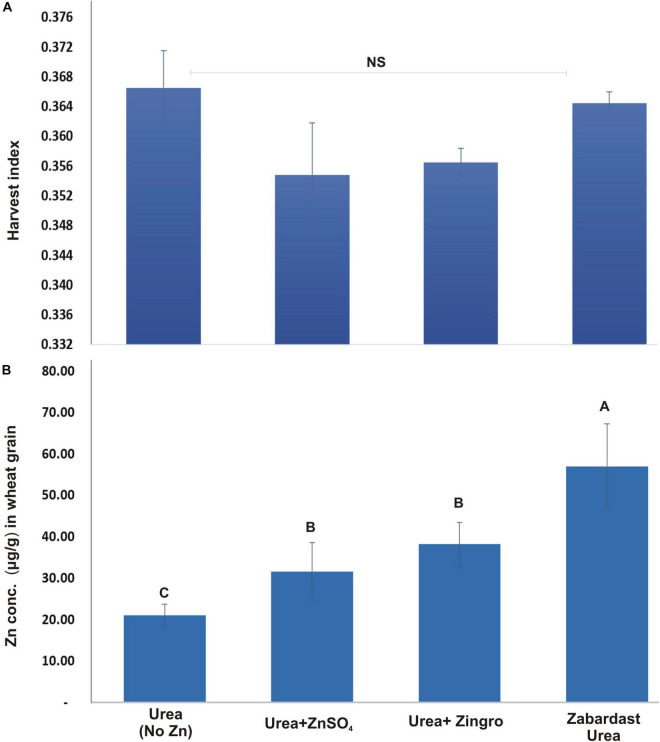
Effect of ZU application on harvest index **(A)** and grain zinc concentration **(B)** of wheat in the field compared with simple urea and Zn treatments. The data are an average of 8 replicated trials (averaged over locations and varieties).

The data for the impact of different Zn treatments on harvest index (HI) from field trials at eight locations is given in Figure 4. The HI data showed a statistically non-significant response of treatments. However, the HI of ZU plots were relatively higher (0.37) than ZnSO_4_ and Zingro (0.35 and 0.36, respectively).

### Zinc Contents in Grains

The statistical analysis of grain Zn contents data obtained from field trials at eight locations (Figure 4) shows that the impact of Zn treatments was statistically significant. ZU application showed the maximum increase in grain Zn contents (57.00 μg/g) with a percentage increase of 171.4 over non-Zn controls. This was followed by Zingro (38.17 μg/g) with a percentage increase of 81.8 and ZnSO_4_ (31.63 μg/g) with an increase of 56% over non-Zn controls (21 μg/g). The average Zn contents in grains of all wheat varieties were maximum in ZU-treated plots at all sites.

### Agronomic Zinc Use Efficiency

The ZUE of different Zn sources used is mentioned in Supplementary Table 3. The data obtained from field trials at eight locations show that ZUE of ZU plots is highest, i.e., 410 followed by the Zingro, i.e., 21, and then ZnSO_4_ with 8.

### Varietal Response Toward Zabardast Urea at Farmer Fields

From the data obtained from farmer field trials (*n* = 119), total yield and Zn contents were measured, and a comparative analysis was made among the ZU vs. farmer practice/check plots. The range of average yield in different varieties in the check plots was 2.67–4.45 t ha^–1^ compared with ZU-treated plots with a yield range of 2.97–4.84 t ha^–1^. An increase in yield with ZU application was observed as a general trend in all 15 varieties when data were averaged over 119 locations, but the yield of 11 varieties showed an increase of more than 10% over the check plots (Supplementary Figure 1A). The varieties, such as Akbar 2019, Ujala, Faisalabad 2008, and Abdul Sattar, showed more than a 20% increase in yield when the data were averaged over locations. The percentage yield increase was less than 10% in only four varieties (Sehar, Punjab 11, Gandum 1, and TD1).

Likewise, average Zn contents of different varieties ranged from 13.00 to 57.33 μg/g with farmer practice while 27.50–69.00 μg/g with ZU treatment (Supplementary Figure 1B). In control plots, except for one variety TJ-83 that showed an inherent potential of high grain Zn contents (57.33 μg/g), all varieties showed Zn contents ≤ 35 μg/g, which are significantly lower than the minimum limit recommended for human consumption. In contrast, ZU treatment substantially increased the grain Zn in all the varieties with a 20–214% increase over the check. Maximum Zn accumulation was observed in a variety Shahbaz that showed 69 μg/g grain Zn, followed by TJ 83 (68.67 μg/g) although it has an inherent ability to accumulate high Zn (Supplementary Figure 1B); wheat variety Al-Ghazi followed with 56 μg/g and Galaxy and Abdul Sattar with ∼46 μg/g Zn in grains. The Zn accumulation in TJ-83 grain shows that it has an inherent ability for Zn accumulation, but the varieties, such as Shahbaz, Al-Ghazi, Sehar, and Ujala, showed an increase in Zn mobilization and accumulation only with the application of ZU.

**FIGURE 5:**
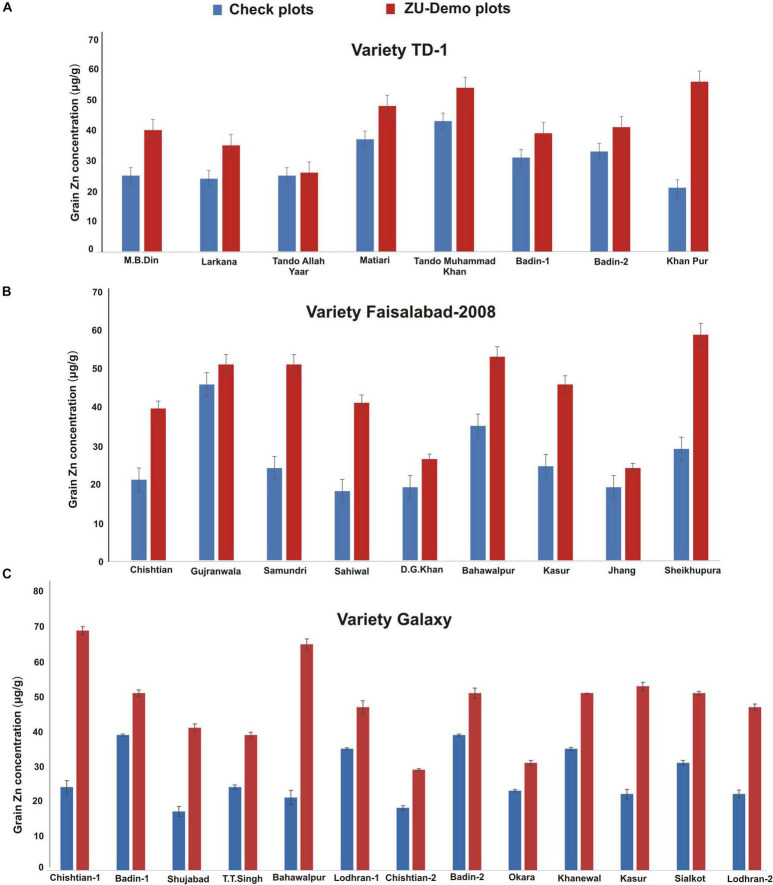
Effect of ZU application on grain Zn concentration of variety TD-1 **(A)**, Faisalabad-2008 **(B)**, and Galaxy **(C)** at different locations in the field compared with check plots.

When data were compared concerning soil (Figure 5), it was observed that Zn contents in wheat varieties vary at different locations/farmer sites. As the varieties recommended at each location/region differ, a general comparison is difficult to draw, but the difference in varietal response may be attributed to the soil conditions, farmer practice, and climate conditions.

### Correlation, Regression, and Interaction Analyses

**FIGURE 6:**
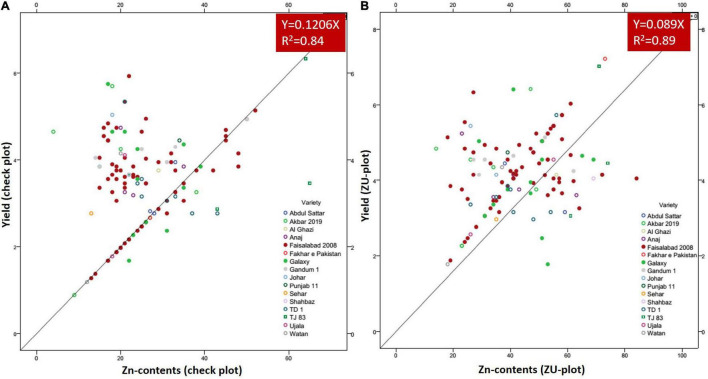
Grain yield response to grain Zn contents in check plots **(A)** and with ZU application **(B)** of different wheat varieties in the field. The data from 8 replicated trials and 111 farmer field demonstration plots have been jointly loaded on the graph to evaluate the response of varieties. The graphs show a positive linear relationship of grain yield and Zn contents in almost all varieties with significantly higher *R*^2^-values.

**FIGURE 7:**
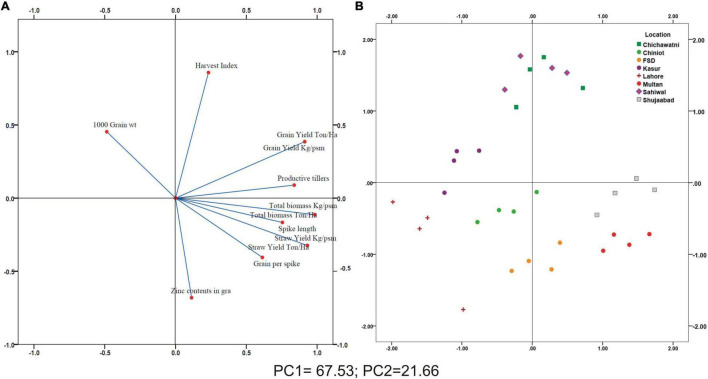
Categorical Principal Component (CAT-PCA) analysis of plant traits measured accross different locations in 15 wheat varieties **(A)** and Principal Component analysis (PCA) showing the joint loading of whole data in a single plot **(B)**; Total variance explained: 89.19%.

**FIGURE 8:**
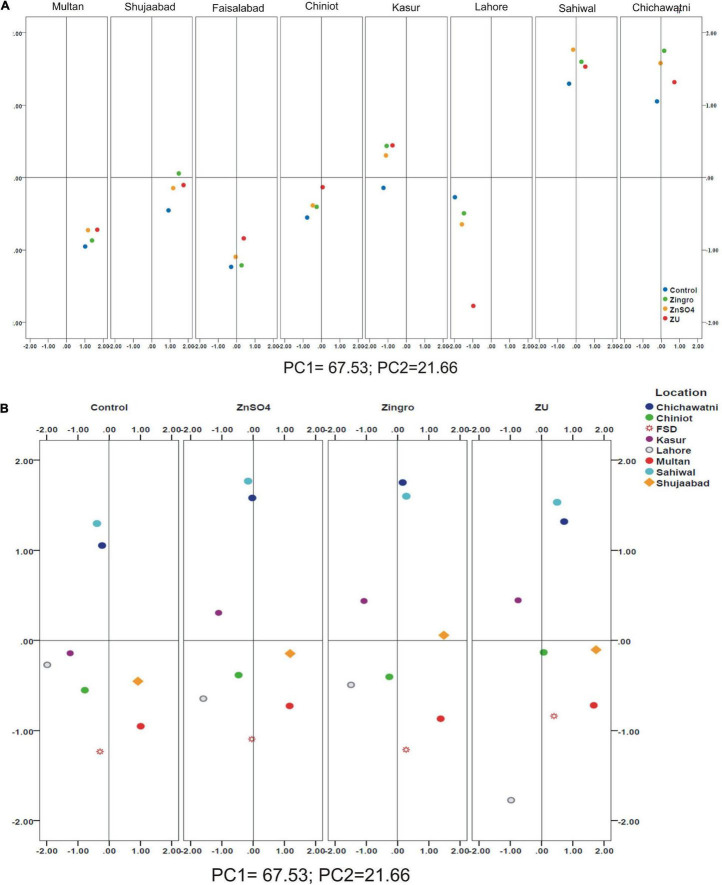
Principal Component (PCA) analysis showing the response of wheat varieties after different Zn-treatments; Loaded location-wise **(A)** and treatment-wise **(B)**.

A positive linear relationship was found among different parameters from the data obtained from farmer fields. Linear regression effectively modeled the positive relationship of grain yield with grain Zn contents, accounting for 65% of the total variance. A positive linear regression (*R*^2^ = 0.83) was observed for yield and Zn contents in the check plots and ZU-treated plots (*R*^2^ = 0.89) (Figures 6A,B). Higher regression value for ZU-treated plots shows the key varietal response toward ZU for Zn uptake and grain accumulation. The CAT-PCA (Figures 7A,B) captured more than 89% of the variance and clearly demonstrated the key environmental differences in the treatment response. The effect of soil was more pronounced (Figure 8A) than the treatments that loaded variably on different quadrants (Figure 8B) in different soils.

## Discussion

A substantial increase in plant nutritional value is very important as billions of people around the globe are suffering from malnutrition effect. Enrichment of staple crops with essential nutrients is of utmost importance to solve the nutrient deficiency issues in humans. This study demonstrates a novel method to improve Zn uptake and accumulation in wheat grains that are inherently deficient in Zn. BNFF is an innovative process that has been used to produce BNFF Urea Z (ZU). The product was tested in countrywide field trials mainly on wheat, which is the main staple crop of Pakistan. The ultimate aim was to use this technology for the wheat biofortification program, which can lead to a decrease in human Zn deficiency in a widespread population.

### Microbe-Mediated Zinc Uptake in Wheat

The ZU exhibited a significantly positive response to facilitate Zn translocation from the rhizosphere to the grains compared with chemical Zn fertilizers in all wheat varieties. Nutrient uptake correlates with grain accumulation and shows a positive impact on plant metabolism and growth (Cakmak and Kutman, 2018; Ahmad et al., 2019). Zn controls several important growth- and yield-regulating processes in plants, e.g., photosynthesis and protein synthesis. It is anticipated that BAZ from ZU made more Zn available for plant uptake. On average, at replicated trials, ZU increased the grain Zn from 21 μg/g (control) to 57 μg/g (171% of the increase), whereas at farmer field demonstration plots ZU increased the grain Zn from 26 μg/g (control) to 44 μg/g (69% of the increase), which is reasonably higher than the desired levels of Zn in wheat grain, i.e., 40 μg/g recommended for human consumption. At farmer fields, the varieties Shahbaz, TJ-83, Al-Ghazi, and Abdul Sattar expressed higher Zn accumulation (>44 μg/g), which can be recommended for the widescale cultivation. Human consumption of this biofortified wheat in a selected population can further validate the implications of ZU technology in terms of reducing malnutrition.

Apart from the beneficial role of BAZ, the ZSB are involved in the increased bioavailability of Zn in plants through the solubilization of insoluble soil Zn fractions present in the rhizosphere. ZSB possibly increased the higher Zn availability during the grain filling stage, which increased the activity of source (flag leaf and stem) and thus more accumulation in grain as previously reported by Cakmak et al. (2010). Enrichment of cereal grains has been reported by ZSB alone and with the combination of organic matter (Hussain et al., 2020). This microbe-mediated grain accumulation of Zn may also cause a reduction in the antinutrient agent, e.g., phytic acid, gluten, tannins, oxalates, lectins, leptins, and saponins, which is helpful to improve the bioavailability of nutrients for human consumption (Vaid et al., 2014; Naz et al., 2016). Phytic acid in grains is not bioavailable and binds to Fe and Zn in grains and makes them unavailable to humans (Thompson, 1989). Reduction in phytic acid accumulation in grains has been related to the increased Zn contents and could be a possible reason for the grain biofortification in this study as reported earlier by Ramesh et al. (2014). The grain phytic acid or the amount of other antinutrients were not determined in this study but may be tested further to see the bioavailability of the accumulated Zn in the grain.

### Improved Zinc Use Efficiency

This study also establishes enhanced “ZUE” as indicated by the active uptake, translocation, and accumulation of Zn in grains of all the wheat varieties. It is established that application of Zn in soil, or as foliar treatment, increases the grain Zn concentration in wheat varieties, e.g., Punjab-11, Faisalabad-2008, and Sehar (Kiran et al., 2021). The translocation and mobilization of Fe and Zn in the grains depending on their concentration in the vegetative tissues of the plant, N status of soil, and nature and type of the plant species or cultivars (Cakmak et al., 2010; Rehman et al., 2018; Singh et al., 2018; Kiran et al., 2021). Our study demonstrates that ZUE of wheat can be increased significantly (up to several folds) by using ZU technology despite 12-folds less Zn (1.235 kg) compared with conventional application practice (15 Kg).

### Impact of Soil and Environment on Zinc Use Efficiency

The available Zn in soil and other nutrient concentration cause a significant impact on the uptake of Zn in the grain. The treatment loading response was variable depending upon the soil condition as evident from the PCA analysis. In Multan, the available P, K, and Zn were relatively lower compared with Shujaabad, which ultimately was reflected on the differential loading of treatments on the PCA plot, although both of these locations are situated in the similar geoclimatic zone. Similarly, Faisalabad and Chiniot, Kasur and Lahore, and Sahiwal and Chichawatni are not very different from each other from a climatic perspective, but the P, K, Zn, B, and SO_4_ available in soil are significantly different, so the treatment response and loading are different.

The data from the farmer fields further show that the ZUE of ZU is higher compared with other Zn recommended forms in all types of soils, i.e., nutrient-sufficient soils or nutrient-deficient soils, and explain the differences in uptake at different locations of the same variety and *vice versa*. The uptake of micronutrients in plants is affected by soil conditions. During farmer field demonstrations at Lodhran, wheat variety Shahbaz showed a yield increase of only 11% but Zn contents were increased up to 214% with ZU application. At the same site Lodhran, another variety Galaxy grown at two locations, the yield increase was 8.9 and 7.9%, while Zn content increase was 34 and 113%, respectively. It has been reported that at farmer fields in Lodhran and Multan, the plant-available Zn and organic matter in the surface soil are 0.1–1.2 mg/kg and 3–17 g/kg, while they are 0.0–0.9 mg/kg and 0–10 g/kg in the sub-surface soil (Maqsood et al., 2014). This high variation in organic matter affects the Zn enrichment of cereal grains (Hussain et al., 2020). A great variation in the nutritional status of Lodhran soil might have responded to this exceptionally high uptake of Zn in grain in this study.

### Other Mechanism at Play During Zinc Solubilization

Widescale field application of ZU demonstrates a significant increase in the growth, yield, and quality of wheat along with a significant increase in the grain Zn contents. ZU outperformed all treatments including chemical Zn fertilizers (farmer practice) at all locations by producing an average yield of 4.06 t ha^–1^, which is 13% higher than controls (3.59 t ha^–1^). This overall increase in plant health and growth could be the synergistic response of BAZ and the supporting activities of microbes, such as P-solubilization, ACC-deaminase activity, production of siderophores, and indole-3-acetic acid potential (Hussain et al., 2015). These microbial traits enhance not only Zn uptake but also other nutrients, establish an extensive root system, and contribute toward better plant health and growth (Whiting et al., 2001). The beneficial microbes produce a variety of organic acids that reduce the pH of the surrounding environment and shift the dynamic equilibrium of minerals from non-labile to labile form which ultimately improves the nutrient uptake, P, Fe, etc., and accumulation in plants (Wani et al., 2007). The microbe-mediated root development and proliferation enhances the capacity of the plant to uptake more nutrients from the soil and provides stronger anchorage to resist lodging at the later stage (Ditta et al., 2018).

It has been recently reported that the application of foliar Zn in wheat varieties Faisalabad-2008, Punjab-11, Saher, and Lasani-2008 shows a significant increase in crop growth rate, plant height, leaf area, total chlorophyll, spikelet per plant, spike length, grains per spike, number of tillers, and productive tillers (Kiran et al., 2021). The Zn application also increased biological yield, harvest index, and grain yield, but the impact was statistically non-significant (Kiran et al., 2021). Similarly, this study reports a statistically significant increase in different growth parameters by application of BAZ, but the impact on harvest index was statistically non-significant. The results have a wide impactful implication of the product to find out the fate of BAZ and beneficial microbes fortified Zn sources in the plant cell *viz.* translocation in the cellular system and the possible interplay in food remobilization during plant growth.

### Economic Analysis

Apart from the application form, Zn nutrition significantly enhanced the benefit-cost ratio. The data show a value to cost ratio (VCR) of 16.0 for ZU, 2.8 for Zingro, and 1.1 for ZnSO_4_ (Supplementary Table 2). This means that spending one rupee will result in a benefit of Rs. 16 with ZU, while 2.8 for Zingro and 1.1 for ZnSO_4_. This cost economics is the most significant factor for the farming community that tends to look for profit maximization. Product profitability is principally associated with the farmer inputs and the yield obtained. Higher VCR for ZU treatment is due to higher yields as well as minimum inputs that subsequently ascertain the monetary benefit to the farmers. As ZU is a urea-coated product, so is more user-friendly as farmers do not need extra application (Zn fertilizer) in the field. It will also contribute significantly in optimizing the cost of Zn to farmers than using Zn separately from the range of chemical products available in the market.

### Consequences for Other Crop Nutrition and Commercialization

The novel BNFF technology has the inherent potential to transform a range of essential plant nutrients into bioactive form. The technology has shown potential and effectiveness in increasing nutrient use efficiency, optimizing the cost of agricultural production, and improving the economics of farmers. The technology has tremendous potentials for future food security and biofortification program of cereals or other agricultural comodities with necessary micronutrients identified as a potential threat to human and animal health not only in Pakistan but in the world at large. The technology can be replicated for cereals, fodders, fruits, and vegetables without changing the fertilizer-application procedure of farmers. The user-friendliness of BNFF technology will help quicker expansion in its application area and crops. The growing use of this technology will continue to contribute positively to improving farmer economics in terms of better yield and quality.

## Conclusion

Countrywide field trials of ZU in bread wheat under varied climatic and soil conditions confirm that ZU is the most effective in increasing grain Zn and ZUE. It has displayed exceptionally consistent results in plant growth, yield, and Zn contents in grains of 15 wheat varieties. The product is biocompatible, user-friendly, and economical for application showing a very high VCR. Keeping in view the emerging public health problems due to Zn deficiency, ZU seems an innovative hybrid solution (biological + chemical) for Zn biofortification, which will help to alleviate Zn deficiency in humans, especially children, and animals at a mass scale without extra efforts and additional cost to the producers or the consumers. To the best of our knowledge, this is the first systematic large-scale field testing of Zn enrichment in the wheat grain of cultivated varieties using this innovative hybrid technology.

## Data Availability Statement

The original contributions presented in the study are included in the article/Supplementary Material, further inquiries can be directed to the corresponding author/s.

## Author Contributions

MA and IA executed the field experiments and did data analysis. FN and NT formulated the product and made the initial draft. AI edited the final draft and data analysis. All authors contributed to the article and approved the submitted version.

## Conflict of Interest

MA and IA were employed by Engro Fertilizers Limited. FN and NT were employed by First Biotech LLC, Lahore. The remaining author declares that the research was conducted in the absence of any commercial or financial relationships that could be construed as a potential conflict of interest.

## Publisher’s Note

All claims expressed in this article are solely those of the authors and do not necessarily represent those of their affiliated organizations, or those of the publisher, the editors and the reviewers. Any product that may be evaluated in this article, or claim that may be made by its manufacturer, is not guaranteed or endorsed by the publisher.
