# Supplementary material for: Bioactive Nutrient Fortified Fertilizer: A Novel Hybrid Approach for the Enrichment of Wheat Grains With Zinc
Source: Front Plant Sci. 2021 Dec 23;12:743378. doi: 10.3389/fpls.2021.743378 (PMC8733721; doi:10.3389/fpls.2021.743378)
Supplement: Supplementary file 1 [file Data_Sheet_1.docx]

**Bioactive Nutrient Fortified Fertilizer (BNFF); a novel hybrid approach for the enrichment of wheat grain with Zinc**

**Muhammad Asif Ali^1^, Farrukh Naeem^2^, Nadeem Tariq^3^, Ijaz Ahmed^1^, Asma Imran^4^**

^1^ Head R&D, Engro Fertilizers Ltd, Lahore

^2^ Director Biotech, First Biotech LLC, Lahore

^3^ Chief Executive, First Biotech LLC, Lahore

^4^ Principal Scientist, National Institute for Biotechnology and Genetic Engineering-Campus-Pakistan Institute for Engineering and Applied Sciences (NIBGE-C-PIEAS), Faisalabad

Corresponding Author

Muhammad Asif Ali

Engro Fertilizers Ltd

Email: [masifali@engro.com](mailto:masifali@engro.com)

| **WHEAT-Experiments At Engro Farms** | | | | | | | | | | |
| --- | --- | --- | --- | --- | --- | --- | --- | --- | --- | --- |
| **Trial #** | **Zone** | **Soil Depth (cm)** | **pH** | **ECe (ds/m)** | **P (ppm)** | **K (ppm)** | **B (ppm)** | **Zn (ppm)** | **SO4 (%)** | **Texture** |
| 1 | **Multan** | 0-15 | 8.2 | 1.62 | 3 | 95 | 0.39 | 0.44 | 0.03 | Loam |
|  |  | 15-30 | 8.5 | 1.31 | 3 | 62 |  |  |  | Loam |
| 2 | **Shujaabad** | 0-15 | 8.2 | 2.91 | 9 | 290 | 0.68 | 1.86 | 0.11 | Loam |
|  |  | 15-30 | 8.0 | 4.38 | 19 | 375 |  |  |  | Loam |
| 3 | **Faisalabad** | 0-15 | 8.4 | 2.46 | 6 | 62 | 0.58 | 0.54 | 0.10 | Loam |
|  |  | 15-30 | 8.4 | 2.20 | 7 | 62 |  |  |  | Loam |
| 4 | **Chiniot** | 0-15 | 8.3 | 2.11 | 5 | 62 | 0.3 | 1.08 | 0.07 | Loam |
|  |  | 15-30 | 8.3 | 2.13 | 6 | 62 |  |  |  | Loam |
| 5 | **Kasur** | 0-15 | 8.2 | 2.90 | 12 | 87 | 0.07 | 1.71 | 0.09 | Loam |
|  |  | 15-30 | 8.0 | 3.18 | 10 | 75 |  |  |  | Loam |
| 6 | **Lahore** | 0-15 | 8.2 | 1.40 | 7.5 | 151 | 0.2 | 0.28 | 0.13 | Loam |
|  |  | 15-30 | 8.3 | 1.31 | 4.9 | 120 | 0.1 | 0.30 | 4.8 | Loam |
| 7 | **Sahiwal** | 0-15 | 8.5 | 4.85 | 5 | 62 | 1.90 | 0.38 | 0.01 | Loam |
|  |  | 15-30 | 8.6 | 3.19 | 5 | 30 |  |  |  | Loam |
| 8 | **Chichawatni** | 0-15 | 8.2 | 1.24 | 13 | 218 | 0.8 | 0.32 | 0.01 | Loam |
|  |  | 15-30 | 8.3 | 1.31 | 7 | 218 |  |  |  | Loam |

**Supplementary Table 1: Detail of soil data from eight locations before testing of ZU at Engro Farms**


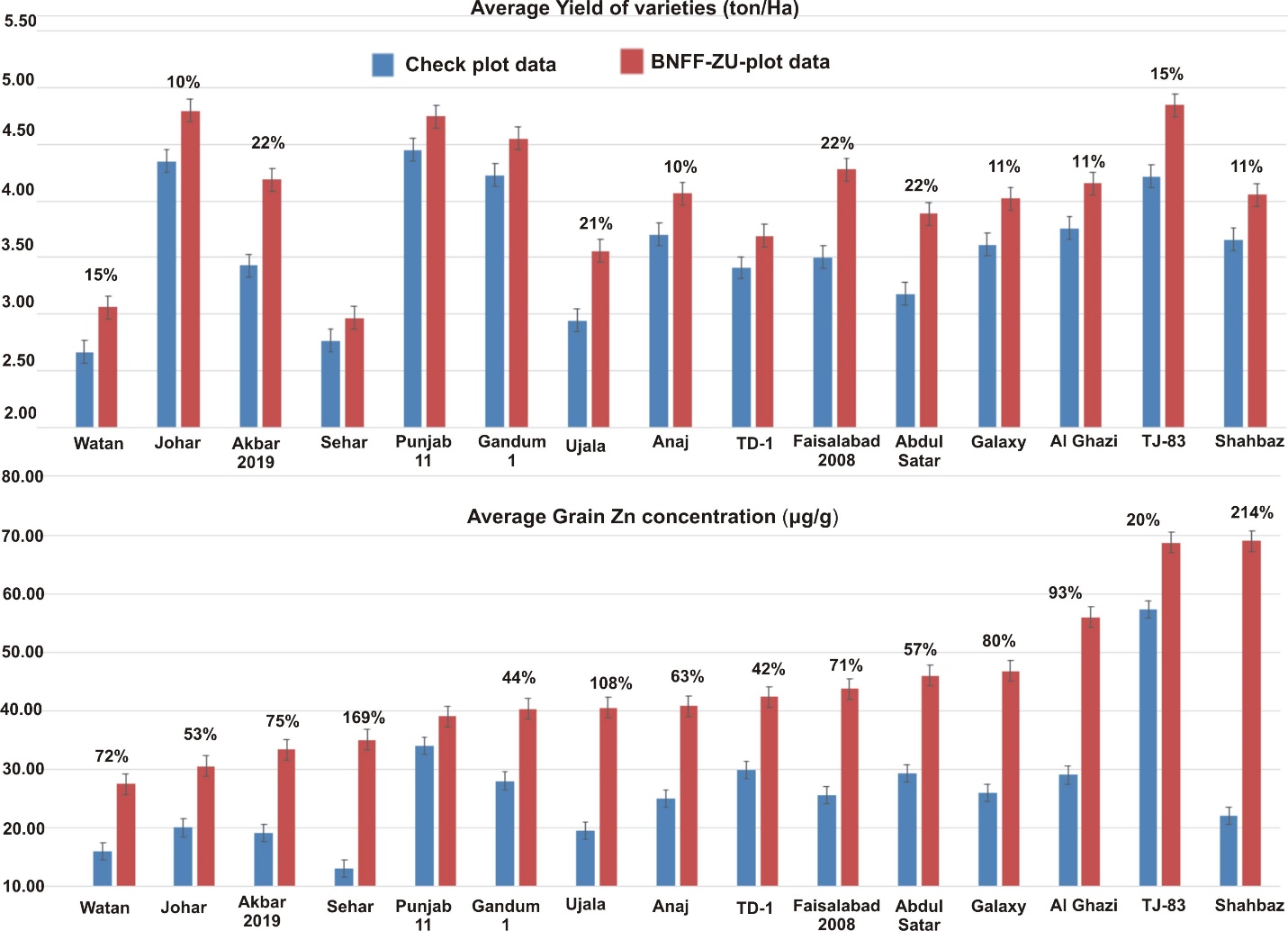


**Supplementary Figure 1: Average yield (A) and Zn contents (B) of 15 wheat varieties at 119 different locations in Farmer demonstration trials**

| **Treatment description** | | **Average Wheat Yield (Kg/Hectare)** | **Increase Yields vs control** | **Zabardast Urea Dose (Kg/ Hectare)** | **Std Urea Dose (Kg/ Hectare)** | **Zinc Dose (Kg/ Hectare)** | **Variable cost (Rs/ Hectare)** | **Incremental Cost (PKR)** | **Incremental Value of Produce (PKR)** | **VCR (Value to Cost Ratio)** |
| --- | --- | --- | --- | --- | --- | --- | --- | --- | --- | --- |
| **T1** | **Standard Urea** | 4363.78 |  | - | 250 | 0 | 6560 |  |  |  |
| **T2** | **Urea + ZnSO_4_** | 4482.39 | 118.6 | - | 250 | 15 | 11601 | 5041 | 5421 | 1.1 |
| **T3** | **Urea + Zingro** | 4677.60 | 313.8 | - | 250 | 15 | 11601 | 5041 | 14158 | 2.8 |
| **T4** | **Zabardust Urea** | 4870.34 | 506.5 | 125 | 125 | 1.235 | 7993 | 1433 | 22878 | 16.0 |

**Supplementary Table 2 (a): The Value to cost ratio of ZU obtained in this study**

**Supplementary Table 2(b): The values used for calculation of VCR in the current studies**

|  | **Price (Rs/pack)** | **Pack size (Kg)** | **Unit Price (Rs/Kg)** |
| --- | --- | --- | --- |

| **Standard Urea Price (2019-20)** | 1770 | 50 | 35 |
| --- | --- | --- | --- |
| **ZU Price (2019-20)** | 2350 | 50 | 47 |
| **Market Zinc (33%) (2019-20)** | 1020 | 3 | 340 |
| **Zingro (33%) (2019-20)** | 1020 | 3 | 340 |
| **Wheat Price (2019-20)** | 1800 | 40 | 45 |

**Supplementary Table 3: Zinc use efficiency of different Zn-treatments**

|  | Yield Kg/Hectare | Zn applied (Kg/Hectare) | Zinc use efficiency |
| --- | --- | --- | --- |
| **Control- Urea** | 4363.78 | 0 |  |
| ZnSO4 | 4482.39 | 15 | 8 |
| Zingro | 4677.60 | 15 | 21 |
| ZU | 4870.34 | 1.235 | 410 |

**Supplementary Table 4: ANOVA tables for eight replicated trials**

| Productive Tillers | | | | | | | *psm* | |  | Statistical Factors | | |
| --- | --- | --- | --- | --- | --- | --- | --- | --- | --- | --- | --- | --- |
| SOV | Deg of freedom | Sum of Squares | Mean Squares | Computed F Value | | F Tabular | | p Value |  | Coefficient of Variance | cov | 3.27% |
|  |  |  |  |  |  | 5% | 1% |  |  | Correction Factor | CF | 4,756,045 |
| Replications | 7 | 49,612.16 | 7,087.45 | 44.71 | ******** | 2.49 | 3.64 | 0.0000 |  | Grand Mean | GM | 385.52 |
| Treatments | 3 | 2,374.06 | 791.35 | 4.99 | ******** | 3.07 | 4.87 | 0.0091 |  | Standard Deviation | Sy | 4.45 |
| Error | 21 | 3,328.65 | 158.51 |  |  |  |  |  |  | LSD 5% |  | 12.99 |
| **Total** | **31** | **55,314.87** |  |  |  |  |  |  |  | LSD 1% |  | 19.23 |
|  |  |  |  |  |  |  |  |  |  |  |  |  |
| Spike Length | | | | | | | *cm* | |  |  | | |
| SOV | Deg of freedom | Sum of Squares | Mean Squares | Computed F Value | | F Tabular | | p Value |  | Coefficient of Variance | cov | 4.02% |
|  |  |  |  |  |  | 5% | 1% |  |  | Correction Factor | CF | 2,879 |
| Replications | 7 | 173.53 | 24.79 | 170.83 | ******** | 2.49 | 3.64 | 0.0000 |  | Grand Mean | GM | 9.49 |
| Treatments | 3 | 3.34 | 1.11 | 7.67 | ******** | 3.07 | 4.87 | 0.0012 |  | Standard Deviation | Sy | 0.13 |
| Error | 21 | 3.05 | 0.15 |  |  |  |  |  |  | LSD 5% |  | 0.39 |
| **Total** | **31** | **179.92** |  |  |  |  |  |  |  | LSD 1% |  | 0.58 |
|  |  |  |  |  |  |  |  |  |  |  |  |  |
| Grains per spike | | | | | | | *Number* | |  |  | | |
| SOV | Deg of freedom | Sum of Squares | Mean Squares | Computed F Value | | F Tabular | | p Value |  | Coefficient of Variance | cov | 4.30% |
|  |  |  |  |  |  | 5% | 1% |  |  | Correction Factor | CF | 74,338 |
| Replications | 7 | 622.27 | 88.90 | 20.73 | ******** | 2.49 | 3.64 | 0.0000 |  | Grand Mean | GM | 48.20 |
| Treatments | 3 | 24.61 | 8.20 | 1.91 | ***ns*** | 3.07 | 4.87 | 0.1584 |  | Standard Deviation | Sy | 0.73 |
| Error | 21 | 90.05 | 4.29 |  |  |  |  |  |  | LSD 5% |  | 2.14 |
| **Total** | **31** | **736.94** |  |  |  |  |  |  |  | LSD 1% |  | 3.16 |
|  |  |  |  |  |  |  |  |  |  |  |  |  |
| 1000 grain weight | | | | | | | *grams* | |  |  |  |  |
| SOV | Deg of freedom | Sum of Squares | Mean Squares | Computed F Value | | F Tabular | | p Value |  | Coefficient of Variance | cov | 2.38% |
|  |  |  |  |  |  | 5% | 1% |  |  | Correction Factor | CF | 34,509 |
| Replications | 7 | 1,137.09 | 162.44 | 265.72 | ******** | 2.49 | 3.64 | 0.0000 |  | Grand Mean | GM | 32.84 |
| Treatments | 3 | 25.65 | 8.55 | 13.98 | ******** | 3.07 | 4.87 | 0.0000 |  | Standard Deviation | Sy | 0.28 |
| Error | 21 | 12.84 | 0.61 |  |  |  |  |  |  | LSD 5% |  | 0.81 |
| **Total** | **31** | **1,175.57** |  |  |  |  |  |  |  | LSD 1% |  | 1.19 |
|  |  |  |  |  |  |  |  |  |  |  |  |  |
| Total Biomass | | | | | | | *Tons per hectare* | |  |  |  |  |
| SOV | Deg of freedom | Sum of Squares | Mean Squares | Computed F Value | | F Tabular | | p Value |  | Coefficient of Variance | cov | 3.14% |
|  |  |  |  |  |  | 5% | 1% |  |  | Correction Factor | CF | 5,320 |
| Replications | 7 | 120.96 | 17.28 | 105.38 | ******** | 2.49 | 3.64 | 0.0000 |  | Grand Mean | GM | 12.89 |
| Treatments | 3 | 10.45 | 3.48 | 21.24 | ******** | 3.07 | 4.87 | 0.0000 |  | Standard Deviation | Sy | 0.14 |
| Error | 21 | 3.44 | 0.16 |  |  |  |  |  |  | LSD 5% |  | 0.42 |
| **Total** | **31** | **134.86** |  |  |  |  |  |  |  | LSD 1% |  | 0.62 |
|  |  |  |  |  |  |  |  |  |  |  |  |  |
| Grain Yield | | | | | | | *Tons per hectare* | |  |  |  |  |
| SOV | Deg of freedom | Sum of Squares | Mean Squares | Computed F Value | | F Tabular | | p Value |  | Coefficient of Variance | cov | 2.04% |
|  |  |  |  |  |  | 5% | 1% |  |  | Correction Factor | CF | 694 |
| Replications | 7 | 27.56 | 3.94 | 438.02 | ******** | 2.49 | 3.64 | 0.0000 |  | Grand Mean | GM | 4.66 |
| Treatments | 3 | 1.17 | 0.39 | 43.44 | ******** | 3.07 | 4.87 | 0.0000 |  | Standard Deviation | Sy | 0.03 |
| Error | 21 | 0.19 | 0.01 |  |  |  |  |  |  | LSD 5% |  | 0.10 |
| **Total** | **31** | **28.92** |  |  |  |  |  |  |  | LSD 1% |  | 0.14 |
|  |  |  |  |  |  |  |  |  |  |  |  |  |
| Straw Yield | | | | | | | *Tons per hectare* | |  |  |  |  |
| SOV | Deg of freedom | Sum of Squares | Mean Squares | Computed F Value | | F Tabular | | p Value |  | Coefficient of Variance | cov | 4.81% |
|  |  |  |  |  |  | 5% | 1% |  |  | Correction Factor | CF | 2,171 |
| Replications | 7 | 52.65 | 7.52 | 47.86 | ******** | 2.49 | 3.64 | 0.0000 |  | Grand Mean | GM | 8.24 |
| Treatments | 3 | 4.96 | 1.65 | 10.53 | ******** | 3.07 | 4.87 | 0.0002 |  | Standard Deviation | Sy | 0.14 |
| Error | 21 | 3.30 | 0.16 |  |  |  |  |  |  | LSD 5% |  | 0.41 |
| **Total** | **31** | **60.92** |  |  |  |  |  |  |  | LSD 1% |  | 0.61 |
|  |  |  |  |  |  |  |  |  |  |  |  |  |
| Harvest Index | | | | | | | *Rato* | |  |  | | |
| SOV | Deg of freedom | Sum of Squares | Mean Squares | Computed F Value | | F Tabular | | p Value |  | Coefficient of Variance | cov | 3.99% |
|  |  |  |  |  |  | 5% | 1% |  |  | Correction Factor | CF | 4 |
| Replications | 7 | 0.04 | 0.01 | 29.81 | ******** | 2.49 | 3.64 | 0.0000 |  | Grand Mean | GM | 0.36 |
| Treatments | 3 | 0.00 | 0.00 | 1.29 | ***ns*** | 3.07 | 4.87 | 0.3025 |  | Standard Deviation | Sy | 0.01 |
| Error | 21 | 0.00 | 0.00 |  |  |  |  |  |  | LSD 5% |  | 0.01 |
| **Total** | **31** | **0.05** |  |  |  |  |  |  |  | LSD 1% |  | 0.02 |
|  |  |  |  |  |  |  |  |  |  |  |  |  |
| Zn Contents in grains | | | | | | | *μg / g* | |  |  | | |
| SOV | Deg of freedom | Sum of Squares | Mean Squares | Computed F Value | | F Tabular | | p Value |  | Coefficient of Variance | cov | 20.09% |
|  |  |  |  |  |  | 5% | 1% |  |  | Correction Factor | CF | 43,690 |
| Replications | 7 | 1,162.38 | 166.05 | 3.01 | ******* | 2.49 | 3.64 | 0.0235 |  | Grand Mean | GM | 36.95 |
| Treatments | 3 | 5,489.44 | 1,829.81 | 33.21 | ******** | 3.07 | 4.87 | 0.0000 |  | Standard Deviation | Sy | 2.62 |
| Error | 21 | 1,157.13 | 55.10 |  |  |  |  |  |  | LSD 5% |  | 7.66 |
| **Total** | **31** | **7,808.95** |  |  |  |  |  |  |  | LSD 1% |  | 11.34 |
